# A Novel Joint Motion Compensation Algorithm for ISAR Imaging Based on Entropy Minimization

**DOI:** 10.3390/s24134332

**Published:** 2024-07-03

**Authors:** Jishun Li, Yasheng Zhang, Canbin Yin, Can Xu, Pengju Li, Jun He

**Affiliations:** Graduate School, Space Engineering University, Beijing 101416, China; lijishun9981@163.com (J.L.); expressesp@126.com (C.Y.); canxu1985@163.com (C.X.); lipengju@nuaa.edu.cn (P.L.); hj1997@stu.xjtu.edu.cn (J.H.)

**Keywords:** inverse synthetic aperture radar (ISAR), space targets, joint motion compensation, entropy minimization, noise robust

## Abstract

Space targets move in orbit at a very high speed, so in order to obtain high-quality imaging, high-speed motion compensation (HSMC) and translational motion compensation (TMC) are required. HSMC and TMC are usually adjacent, and the residual error of HSMC will reduce the accuracy of TMC. At the same time, under the condition of low signal-to-noise ratio (SNR), the accuracy of HSMC and TMC will also decrease, which brings challenges to high-quality ISAR imaging. Therefore, this paper proposes a joint ISAR motion compensation algorithm based on entropy minimization under low-SNR conditions. Firstly, the motion of the space target is analyzed, and the echo signal model is obtained. Then, the motion of the space target is modeled as a high-order polynomial, and a parameterized joint compensation model of high-speed motion and translational motion is established. Finally, taking the image entropy after joint motion compensation as the objective function, the red-tailed hawk–Nelder–Mead (RTH-NM) algorithm is used to estimate the target motion parameters, and the joint compensation is carried out. The experimental results of simulation data and real data verify the effectiveness and robustness of the proposed algorithm.

## 1. Introduction

Inverse synthetic aperture radar (ISAR) is an important sensor for the observation and imaging of aerial and space targets. Compared with optical sensor, ISAR is free from the interference of sky background light and cloud occlusion, has better all-weather working ability, and has a long detection distance, so it plays an important role in space target surveillance [1,2,3,4]. The range resolution of ISAR depends on the radar bandwidth, and the azimuth resolution depends on the relative motion between the target and the radar [5,6,7]. The target of interest for ISAR is non-cooperative, meaning that the motion parameters is unknown in advance, which poses a significant challenge to high-quality ISAR imaging [8]. Generally speaking, the motion of the target relative to the radar can be divided into two parts: translational motion and rotation motion [9,10]. The rotational motion provides the azimuth resolution that is needed for imaging, while the translational motion will cause range cell misalignment and phase error, which make the ISAR image defocused and blurry. Therefore, in order to achieve high-quality ISAR imaging, the translational motion needs to be compensated for [11].

The key to high-quality ISAR imaging lies in the precise compensation for translational motion. Current methods of translational motion compensation (TMC) are divided into two main categories. One category is non-parametric TMC methods, also known as adjacent TMC methods, which are carried out in two steps: range alignment (RA) [12,13,14] and phase adjustment (PA) [15,16,17,18]. RA can eliminate range cell misalignment. The resemblance between adjacent range profiles is utilized to find the correct number of range cells for shifting each range profile [19]. Apart from resemblance-based methods, there is another type of RA algorithm that relies on optimizing the quality measures of the alignment. The methods based on the entropy and the contrast of the average range profile are presented in [20] and [21], respectively. After RA, PA is utilized to eliminate phase error. The algorithms for PA mainly include the following: the dominant scatters algorithm [22], the Doppler centroid tracking (DCT) algorithm [23], the phase gradient autofocus (PGA) algorithm [24], and the eigenvector-based algorithm [25]. There are also algorithms based on image quality assessment, such as the minimum entropy algorithm [26,27,28] and the maximum contrast algorithm [29,30,31].

Another category is parametric methods, also referred to as joint TMC methods [32,33,34,35]. These methods utilize entropy or contrast as the focus quality assessment metrics for ISAR images. Subsequently, the motion of the target is modeled as a high-order polynomial, and the optimal image focus quality assessment metrics are used to solve for the motion parameters of the target. Then, range cell misalignment and phase error are compensated for simultaneously. This compensation method does not depend on the resemblance between adjacent range profiles, thus avoiding the impact of residual RA error on PA. Therefore, it can achieve better TMC even under low signal-to-noise ratio (SNR) conditions.

It is noteworthy that the above-mentioned TMC methods are all aimed at low-speed targets such as airplanes and ships. For high-speed moving targets such as satellites and missiles, the motion velocity is usually several kilometers per second, and the “stop-go” model is no longer applicable. The high resolution range profile (HRRP) will be stretched due to the high-speed motion of the target, which affects the accuracy of RA, thereby affecting subsequent PA and the ISAR image would be seriously blurred [36]. Therefore, before performing TMC, it is necessary to estimate the velocity of the target and carry out high-speed motion compensation (HSMC).

The current HSMC methods are divided into two main categories. One category is based on signal parameter estimation, which models the echo of each pulse as a multi-component higher-order phase signal and then estimates the signal parameters through fractional Fourier transform (FrFT) [37,38], integrating cubic phase functions (ICPF) [39], etc., and further obtaining the target motion velocity. This category relies on the accurate estimation of signal parameters and is easily affected by noise. The other category is based on the focusing quality of HRRP [40,41], constructing compensation terms with different speeds to compensate for the echo. The target motion velocity is estimated by optimizing the focusing quality of HRRP. Waveform entropy and contrast are both commonly used focusing quality assessment metrics. The limitation of these algorithms lies in the fact that they process each pulse independently, resulting in a large error in velocity estimation. Owing to the separate processing of echoes, within one coherent processing interval (CPI), the high-speed motion estimation error of each pulse gradually accumulates, leading to a poor overall high-speed compensation effect.

In ISAR imaging, due to transmission loss or limitations in the transmitted energy, the issue of a low SNR of the target echo often occurs. In the case of a low SNR, both HSMC and TMC face challenges [42,43]. Wang [44] proposed an HSMC method based on the minimum entropy of two-dimensional ISAR images, which achieves high-quality imaging under low SNRs. However, this method assumes that the translational error can be eliminated through TMC, leaving only the high-speed motion error in the echo. In practice, due to the presence of high-speed motion error, TMC becomes very difficult. Therefore, the assumption of this method is overly idealized, which significantly limits its applicability. In the case of low SNR, the precision of HSMC diminishes, and the residual error of HSMC will lead to a decrease in the precision of TMC, which severely degrades the quality of ISAR imaging.

Aiming to perform high-quality ISAR imaging of space targets under a low SNR, a noise-robust joint motion compensation algorithm for ISAR imaging-based entropy minimization is proposed in this paper. Firstly, the influence of the high-speed motion and translational motion of the space targets on the echo in de-chirp mode is analyzed, and the signal model of the space targets is established. Considering the continuity of the motion of the target in a CPI, the motion of the target is modeled as a high-order polynomial, and the motion polynomial coefficients are optimized by minimizing two-dimensional image entropy. Based on the established minimum entropy optimization model, the red-tailed hawk–Nelder–Mead (RTH-NM) algorithm is used to solve the minimum entropy optimization problem, and then joint motion compensation is realized. Electromagnetic simulation data and Yak-42 measured data verify the effectiveness of the joint motion compensation algorithm. Compared with existing algorithms, this algorithm is innovative in the following aspects:

- A novel joint compensation model for the simultaneous compensation of high-speed motion and translational motion is proposed for the first time. Existing methods typically separate HSMC and TMC into two steps. The residual error from HSMC will affect the accuracy of TMC. However, in this paper, a parametric joint compensation model is used to simultaneously compensate for the high-speed motion and translation motion of the target. The joint motion compensation reduces the impact of residual error from HSMC on TMC, thus achieving higher motion compensation accuracy.
- Many existing parametric motion compensation methods rely on gradient-based approaches to solve problems, which necessitate intricate derivative calculations and are highly sensitive to the selection of initial values. In this paper, a two-step optimization method that synergizes the red-tailed hawk (RTH) algorithm with the Nelder–Mead (NM) algorithm, called the RTH-NM algorithm, is used to estimate the target motion parameters. The RTH algorithm facilitates the avoidance of local optima during parameter optimization, enabling a preliminary search for parameters. The NM algorithm, on the other hand, achieves a more refined search, ensuring the precision of motion parameters. The integration of both algorithms enables rapid convergence towards an accurate solution, identifying the optimal motion parameters. In comparison to gradient-based methods, this approach proves to be more effective and pragmatic.
- This algorithm fully utilizes the high SNR gain accumulated from two-dimensional ISAR images, which is beneficial for the joint compensation of high-speed motion and translation motion under low-SNR conditions. It improves the accuracy of motion compensation, leading to the enhanced quality of ISAR images.

This study is based on the following assumptions: (1) Random disturbances in the envelope caused by the radar system and the changing sampling wave gate are not considered. (2) Within the imaging CPI, the relative rotation angle of the target is small, and the equivalent rotation angular velocity of the target is constant.

This paper is organized as follows. Section 2 introduces the de-chirp signal mode for space targets. In Section 3, a joint motion compensation model based on entropy minimization is established, and the RTH-NM algorithm is used to estimate the target motion parameters. In Section 4, the experimental results of simulation data and real data are given, and the effectiveness and robustness of the proposed algorithm are analyzed. Finally, some conclusions are summarized in Section 5.

## 2. De-Chirp Signal Model for Space Targets

**Figure 1:**
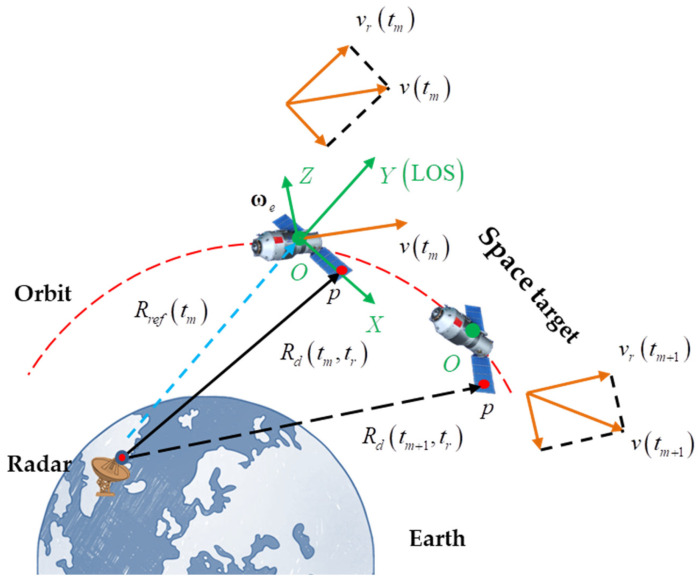
Observation geometry for space target.

The imaging geometric configuration of the radar and space target is shown in Figure 1. O is the origin of the coordinate system, located at the centroid of the space target. The direction of the radar line of sight (LOS) corresponds to the Y-axis. Assuming the effective rotational vector (ERV) of the target is $\omega_{e}$, then the direction of $\omega_{e}$ is the Z-axis of the coordinate system. The X-axis of the coordinate system can be obtained by the right-hand rule. The XOY plane is the image projection plane (IPP). Supposing that the wideband radar transmits a linear frequency modulated (LFM) signal,

(1) $$s(t_{r},t_{m})=\mathrm{rect}\left(\frac{t_{r}}{T_{P}}\right)\exp\left(j2\pi f_{c}t\right)\exp\left(j\pi \gamma t_{r}{\;\;}^{2}\right)$$

where $\mathrm{rect}\left(\frac{t_{r}}{T_{p}}\right)=\left\{\begin{array}{c} 1,\\ 0, \end{array}\begin{array}{c} \;\\ \; \end{array}\right.\begin{array}{c} \left|t_{r}/T_{p}\right|\leq 0.5\\ \left|t_{r}/T_{p}\right|>0.5 \end{array}$, and $T_{p}$, $f_{c}$, and γ represent pulse width, carrier frequency, and frequency modulation rate, respectively. $t=t_{r}+t_{m}$ is the full time, where $t_{r}$ is the fast time and $t_{m}$ is the slow time.

As shown in Figure 1, assuming that the space target consists of P scattering centers and p is an arbitrary scattering center, the radar echo of p can be written as

(2) $$s_{p}(t_{r},t_{m})=\sigma_{p}\mathrm{rect}\left(\frac{t_{r}-t_{d}}{T_{p}}\right)\exp\left(j2\pi f_{c}\left(t-t_{d}\right)\right)\exp\left(j\pi \gamma {\left(t_{r}-t_{d}\right)}^{2}\right)$$

where $t_{d}=2R_{\mathrm{dp}}\left(t_{m}\right)/c$ is the echo time delay of p, $R_{\mathrm{dp}}\left(t_{m}\right)$ is the instantaneous distance from p to radar at $t_{m}$, c is the propagation speed of light, and $\sigma_{p}$ is the backscattering coefficient of p. Due to the high-speed motion of the space target, the distance change of the target within one pulse width needs to be considered. So, the distance from p to the radar can be rewritten as

(3) $$R_{\mathrm{dp}}\left(t_{r},t_{m}\right)=R_{\mathrm{dp}1}\left(t_{m}\right)+R_{\mathrm{dp}2}\left(t_{r}\right)$$

where $R_{\mathrm{dp}1}\left(t_{m}\right)$ is the distance change with $t_{m}$, and $R_{\mathrm{dp}2}\left(t_{r}\right)$ is the distance change with $t_{r}$. Considering the short duration of a pulse, the variation in velocity within a pulse can be neglected. That is, if the target can be approximated as moving at a constant speed within a pulse, then $R_{\mathrm{dp}2}\left(t_{r}\right)$ can be approximated as

(4) $$R_{\mathrm{dp}2}\left(t_{r}\right)\approx v\left(t_{m}\right)\cdot t_{r}$$

where $v\left(t_{m}\right)$ is the radial velocity of the target at $t_{m}$. By taking the conjugate multiplication of the echo signal with the reference signal, de-chirp processing can be carried out. The reference signal is

(5) $$s_{ref}(t_{r},t_{m})=\mathrm{rect}\left(\frac{t_{r}-t_{ref}}{T_{ref}}\right)\exp\left(j2\pi f_{c}\left(t-t_{ref}\right)\right)\exp\left(j\pi \gamma {\left(t_{r}-t_{ref}\right)}^{2}\right)$$

where $t_{ref}=2R_{ref}\left(t_{m}\right)/c$, $R_{ref}\left(t_{m}\right)$ is the reference distance at $t_{m}$. After de-chirp processing, we can obtain the output signal as follows:

(6) $$\begin{array}{c} s(t_{r},t_{m})=s_{p}(t_{r},t_{m})\cdot s_{{\;\;}_{ref}}^{*}(t_{r},t_{m})\\ \;=\sigma_{p}\mathrm{rect}\left(\frac{t_{r}-t_{d}}{T_{p}}\right)\cdot rect\left(\frac{t_{r}-t_{ref}}{T_{ref}}\right)\\ \;\cdot \exp\left\{-j2\pi \left[f_{c}\left(t_{d}-t_{ref}\right)+\gamma t_{r}\left(t_{d}-t_{ref}\right)-\frac{1}{2}\gamma \left(t_{d}^{2}-t_{ref}^{2}\right)\right]\right\}\\ \;=\sigma_{p}\mathrm{rect}\left(\frac{t_{r}-t_{d}}{T_{p}}\right)\cdot \exp\left(j\frac{4\pi \gamma }{c^{2}}\Delta r_{p}{\;\;}^{2}\right)\\ \;\cdot \exp\left(-j\frac{4\pi }{c}\left(f_{c}+\gamma \left(t_{r}-t_{ref}\right)\right)\Delta r_{p}\right) \end{array}$$

where $\Delta r_{p}=R_{\mathrm{dp}}\left(t_{r},t_{m}\right)-R_{ref}\left(t_{m}\right)$. For the sake of conciseness, let $t_{r}=t_{r}-t_{ref}$ represent the new fast time. Then, Equation (6) can be rewritten as

(7) $$\begin{array}{c} s(t_{r},t_{m})=\sigma_{p}\mathrm{rect}\left(\frac{t_{r}-2\Delta r_{p}/c}{T_{p}}\right)\cdot \exp\left(j\frac{4\pi \gamma }{c^{2}}\Delta r_{p}{\;\;}^{2}\right)\\ \cdot \exp\left(-j\frac{4\pi }{c}\left(f_{c}+\gamma t_{r}\right)\Delta r_{p}\right) \end{array}$$

Assuming that the coordinate of p in the imaging plane XOY is $\left(x_{p},y_{p}\right)$, and considering that the relative rotation angle of the target within the CPI is small and the rotation is usually uniform, the instantaneous distance from p to radar is given by

(8) $$\begin{array}{c} R_{\mathrm{dp}1}\left(t_{m}\right)=R_{T}\left(t_{m}\right)+x_{p}\sin\left(\omega t_{m}\right)+y_{p}\cos\left(\omega t_{m}\right)\\ \approx R_{T}\left(t_{m}\right)+x_{p}\omega t_{m}+y_{p} \end{array}$$

where $R_{T}\left(t_{m}\right)$ is the translational motion of the target centroid, ω is the angular velocity, and $\omega =\left|\omega_{e}\right|$. Substituting Equation (8) into Equation (7) and performing the Taylor expansion yields

(9) $$\begin{array}{l} s\left(t_{r},t_{m}\right)=\sigma_{p}\mathrm{rect}\left(\frac{t_{r}-2\Delta r_{p}/c}{T_{p}}\right)\cdot \exp\left(-j\frac{4\pi f_{c}}{c}\left(R_{T}\left(t_{m}\right)-R_{ref}\left(t_{m}\right)\right)\right)\\ \;\cdot \exp\left(-j\frac{4\pi \gamma t_{r}}{c}\left(R_{T}\left(t_{m}\right)-R_{ref}\left(t_{m}\right)\right)\right)\\ \;\cdot \exp\left(-j\frac{4\pi f_{c}}{c}x_{p}\omega t_{m}\right)\cdot \exp\left(-j\frac{4\pi \gamma t_{r}}{c}x_{p}\omega t_{m}\right)\\ \;\cdot \exp\left(-j\frac{4\pi f_{c}}{c}y_{p}\right)\cdot \exp\left(-j\frac{4\pi \gamma t_{r}}{c}y_{p}\right)\\ \;\cdot \exp\left(j\frac{4\pi v\left(t_{m}\right)}{c}\left(\frac{2\gamma R_{\Delta }}{c}-f_{c}\right)\cdot t_{r}\right)\\ \;\cdot \exp\left(j\frac{4\pi \gamma v\left(t_{m}\right)}{c}\left(\frac{v\left(t_{m}\right)}{c}-1\right)t_{r}{\;\;}^{2}\right)\\ \;\cdot \exp\left(j\frac{4\pi \gamma }{c^{2}}R_{\Delta }{\;\;}^{2}\right) \end{array}$$

where $R_{\Delta }=R_{T}\left(t_{m}\right)-R_{ref}\left(t_{m}\right)+x_{p}\omega t_{m}+y_{p}$ represents the distance of p from the reference point at $t_{m}$, and the phase in Equation (9) can be divided into nine terms. The first term $\exp\left(-j\frac{4\pi f_{c}}{c}\left(R_{T}\left(t_{m}\right)-R_{ref}\left(t_{m}\right)\right)\right)$ is the phase error term, and the second term $\exp\left(-j\frac{4\pi \gamma t_{r}}{c}\left(R_{T}\left(t_{m}\right)-R_{ref}\left(t_{m}\right)\right)\right)$ is the range cell misalignment term; both of them are caused by the translational motion of the target. The third term $\exp\left(-j\frac{4\pi f_{c}}{c}x_{p}\omega t_{m}\right)$ is the rotational Doppler term of p, which is the source of the ISAR azimuth resolution. The fourth term $\exp\left(-j\frac{4\pi \gamma t_{r}}{c}x_{p}\omega t_{m}\right)$ is the range migration term caused by rotational motion, which usually does not exceed a range cell in ISAR imaging, and its impact can be negligible. The fifth term $\exp\left(-j\frac{4\pi f_{c}}{c}y_{p}\right)$ is constant and can be ignored. The sixth term $\exp\left(-j\frac{4\pi \gamma t_{r}}{c}y_{p}\right)$ is the range compression term of p. The seventh term $\exp\left(j\frac{4\pi v\left(t_{m}\right)}{c}\left(\frac{2\gamma R_{\Delta }}{c}-f_{c}\right)\cdot t_{r}\right)$ is the envelope walk term, and the eighth term $\exp\left(j\frac{4\pi \gamma v\left(t_{m}\right)}{c}\left(\frac{v\left(t_{m}\right)}{c}-1\right)t_{r}{\;\;}^{2}\right)$ is the range profile stretched term; they are all caused by the target’s high-speed motion. The ninth term $\exp\left(j\frac{4\pi \gamma }{c^{2}}R_{\Delta }{\;\;}^{2}\right)$ is the residual video phase (RVP) error, which can be removed by RVP compensation. From Equation (9), we can discover that high-speed motion leads to envelope walk and a stretched range profile. Due to the reduction in similarity between HRRP caused by the stretch of the range profile, the accuracy of the adjacent TMC method will decrease. Simultaneously, the high-speed motion of the target also causes envelope walk, which breaks the homology between the envelope walk and the phase error of translation motion and will also reduce the effectiveness of parametric TMC. Under low-SNR conditions, the impact of high-speed motion will be more significant, which may lead to the failure of traditional motion compensation methods and the inability to achieve ISAR imaging.

Subsequently, the seventh phase term of (9) is further analyzed and written as

(10) $$\exp\left(j\frac{4\pi v\left(t_{m}\right)}{c}\left(\frac{2\gamma R_{\Delta }}{c}-f_{c}\right)t_{r}\right)={ℜ}_{1}\cdot {ℜ}_{2}$$

where ${ℜ}_{1}=\exp\left(j\frac{4\pi }{c}\left(\frac{2\gamma R_{\Delta }v\left(t_{m}\right)}{c}t_{r}\right)\right)$ and ${ℜ}_{2}=\exp\left(-j\frac{4\pi }{c}f_{c}v\left(t_{m}\right)t_{r}\right)$. Since both ${ℜ}_{1}$ and ${ℜ}_{2}$ are related to the fast time $t_{r}$, both of which will cause envelope walk, the echo from the same scattering point will be distributed across different range cells within different pulses. Therefore, their impact needs to be analyzed.

${ℜ}_{1}$ is related to the position of each scattering point p. The frequency resolution in the range frequency domain is $\Delta f=f_{s}/N$, where N represents the total number of range cells, and $f_{s}$ is the sampling frequency. The range cell offset $\Delta R_{cell1}$ caused by ${ℜ}_{1}$ can be expressed as

(11) $$\begin{array}{c} \Delta R_{cell1}=\frac{4\gamma R_{\Delta }v\left(t_{m}\right)}{c^{2}}/\Delta f\\ =\frac{4\gamma R_{\Delta }v\left(t_{m}\right)N}{c^{2}f_{s}} \end{array}$$

${ℜ}_{2}$ is independent of the position of the scattering point p, and the range cell offset $\Delta R_{cell2}$ caused by ${ℜ}_{2}$ can be expressed as

(12) $$\begin{array}{c} \Delta R_{cell2}=\frac{-2f_{c}v\left(t_{m}\right)}{c}/\Delta f\\ =\frac{-2f_{c}v\left(t_{m}\right)N}{cf_{s}} \end{array}$$

The ratio of the range cell offset caused by ${ℜ}_{1}$ and ${ℜ}_{2}$ is

(13) $$\eta_{cell}=\left|\Delta R_{cell1}/\Delta R_{cell2}\right|=\frac{2\gamma R_{\Delta }}{f_{c}c}$$

**Figure 2:**
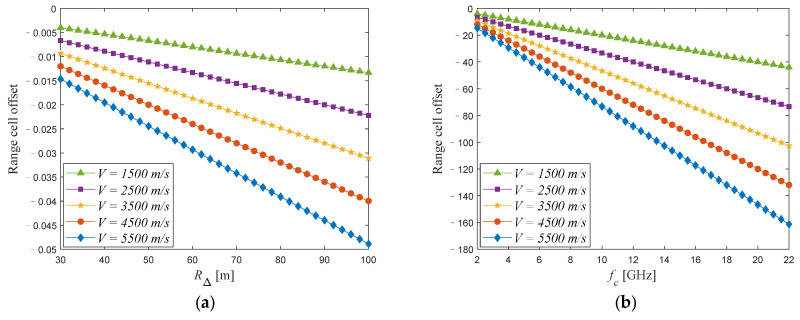

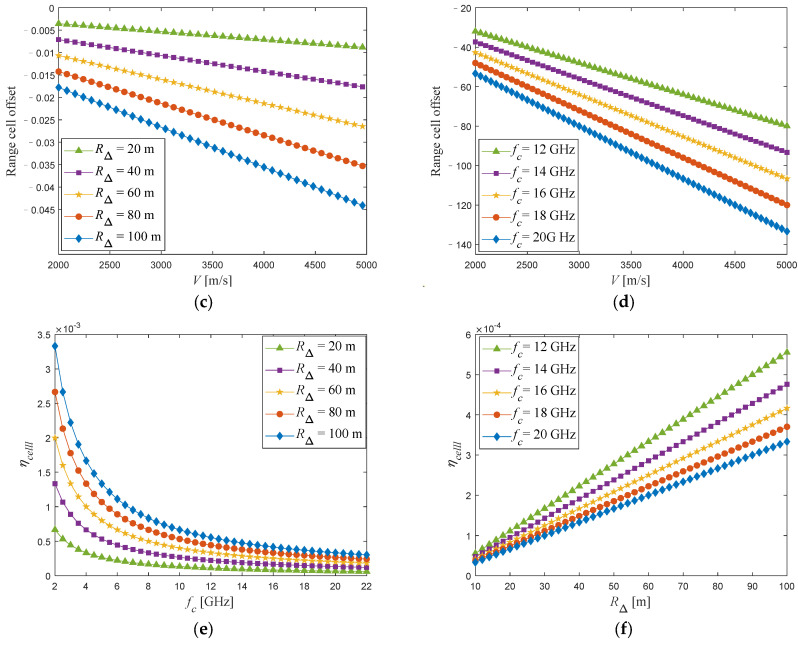
Range cell offset analysis with different factors. (**a**) Variation in $\Delta R_{cell1}$ with $R_{\Delta }$ under different V. (**b**) Variation in $\Delta R_{cell2}$ with $f_{c}$ under different V. (**c**) Variation in $\Delta R_{cell1}$ with V under different $R_{\Delta }$. (**d**) Variation in $\Delta R_{cell2}$ with V under different $f_{c}$. (**e**) Variation in $\eta_{cell}$ with $f_{c}$ under different $R_{\Delta }$. (**f**) Variation in $\eta_{cell}$ with $R_{\Delta }$ under different $f_{c}$.

Subsequently, a simulation analysis is conducted. In the simulation, the frequency modulation rate γ is set to $10^{13}\;\mathrm{Hz}/s$. Considering that the maximum size of the space target currently observed is within one hundred meters, we set the maximum of $R_{\Delta }$ to 100 m. V is the velocity of space target. The simulation results are shown in Figure 2.

As can be seen from Figure 2, the range cell offset caused by ${ℜ}_{1}$ is basically less than 0.05 range cells, while the range cell offset caused by ${ℜ}_{2}$ can reach several tens or even hundreds of range cells, and $\eta_{cell}$ is maintained at the order of magnitude of 10^−4^ when $f_{c}$ is greater than 10 GHz. That is to say, the range cell offset is mainly affected by ${ℜ}_{2}$; therefore, we ignore ${ℜ}_{1}$. Similar conclusions can also be found in [45]. At the same time, we can observe that when the target’s velocity changes to about 400 m/s, the variation in $\Delta R_{cell2}$ can reach approximately 10 range cells. It is precisely because of the variation in $\Delta R_{cell2}$ that the homology between range cell misalignment and phase error is disrupted, and the joint TMC method will become unusable as a result. After the above analysis, we can obtain the final space target echo signal as follows:

(14) $$\begin{array}{l} s\left(t_{r},t_{m}\right)=\tilde{s}\left(t_{r},t_{m}\right)\cdot \exp\left[-j\frac{4\pi }{c}f_{c}\left(R_{T}\left(t_{m}\right)-R_{ref}\left(t_{m}\right)\right)\right]\\ \;\cdot \exp\left[-j\frac{4\pi }{c}\gamma \left(R_{T}\left(t_{m}\right)-R_{ref}\left(t_{m}\right)\right)t_{r}\right]\\ \;\cdot \exp\left[j\frac{4\pi \gamma v\left(t_{m}\right)}{c}\left(\frac{v\left(t_{m}\right)}{c}-1\right)t_{r}^{2}\right]\\ \;\cdot \exp\left[-j\frac{4\pi f_{c}v\left(t_{m}\right)}{c}t_{r}\right] \end{array}$$

where $\tilde{s}\left(t_{r},t_{m}\right)$ is the space target echo of the ideal turntable model and can be represented as

(15) $$\tilde{s}\left(t_{r},t_{m}\right)=\sigma_{p}\mathrm{rect}\left(\frac{t_{r}-2\Delta r_{p}/c}{T_{p}}\right)\cdot \exp\left(-j\frac{4\pi f_{c}}{c}x_{p}\omega t_{m}\right)\cdot \exp\left(-j\frac{4\pi \gamma t_{r}}{c}y_{p}\right)$$

According to Equation (14), the signal model for joint compensation for high-speed motion and translational motion is

(16) $$\begin{array}{l} \tilde{s}\left(t_{r},t_{m}\right)=s\left(t_{r},t_{m}\right)\cdot \exp\left[j\frac{4\pi }{c}f_{c}\left(R_{T}\left(t_{m}\right)-R_{ref}\left(t_{m}\right)\right)\right]\\ \;\cdot \exp\left[j\frac{4\pi }{c}\gamma \left(R_{T}\left(t_{m}\right)-R_{ref}\left(t_{m}\right)\right)t_{r}\right]\\ \;\cdot \exp\left[-j\frac{4\pi \gamma v\left(t_{m}\right)}{c}\left(\frac{v\left(t_{m}\right)}{c}-1\right)t_{r}^{2}\right]\\ \;\cdot \exp\left[j\frac{4\pi }{c}\left(f_{c}v\left(t_{m}\right)\right)t_{r}\right] \end{array}$$

By discretizing the echo and performing the fast Fourier transform (FFT) with respect to $t_{r}$ and $t_{m}$, the ISAR image after joint motion compensation can be expressed as

(17) $$\begin{array}{l} I\left(k,h\right)=\sum_{m=1}^{M}\exp\left(-j2\pi \frac{hm}{M}\right)\sum_{n=1}^{N}\exp\left(-j2\pi \frac{kn}{N}\right)\\ \;\cdot s\left(n,m\right)\cdot \exp\left[j\frac{4\pi }{c}f_{c}\left(R_{T}\left(m\right)-R_{ref}\left(m\right)\right)\right]\\ \;\cdot \exp\left[j\frac{4\pi }{c}\gamma \left(R_{T}\left(m\right)-R_{ref}\left(m\right)\right)n\right]\\ \;\cdot \exp\left[-j\frac{4\pi \gamma v\left(m\right)}{c}\left(\frac{v\left(m\right)}{c}-1\right)n^{2}\right]\\ \;\cdot \exp\left[j\frac{4\pi }{c}\left(f_{c}v\left(m\right)\right)n\right]+\xi \left(k,h\right) \end{array}$$

where $I\left(k,h\right)$ is the ISAR image after compensation. k and h are the serial numbers of the range cells and Doppler cells, respectively. k=1,2,⋅⋅⋅,N and h=1,2,⋅⋅⋅,M, where N is the number of range cells and M is the number of Doppler cells. $s\left(n,m\right)$ is the discrete form of $s\left(t_{r},t_{m}\right)$. n and m are the discrete form of $t_{r}$ and $t_{m}$. $\xi \left(k,h\right)$ denotes complex noise. Equation (17) is the signal model of the final ISAR images after joint motion compensation. In the following sections, the joint motion compensation algorithm based on parametric minimum entropy optimization uses this signal model.

## 3. Optimization of Joint Motion Compensation

### 3.1. Optimization of Motion Parameters Based on Minimum Entropy

Based on the joint motion compensation model established in the previous subsection, joint motion compensation can be achieved if the motion parameters of the target can be accurately estimated. Therefore, the key issue lies in how to accurately estimate the motion parameters of the target. Unlike aircraft and vessels, space targets typically move along orbital trajectories utilizing a three-axis stabilization mode, and the motion is relatively stable. Without the loss of generality, the radial motion of the space target can be model as an L-order polynomial:

(18) $$R_{T}\left(m\right)=\sum_{l=0}^{L}b_{l}{\left(m\Delta t_{m}\right)}^{l}$$

and the radial velocity of the space target can also be expressed as

(19) $$v\left(m\right)=\sum_{l=1}^{L}l\cdot b_{l}{\left(m\Delta t_{m}\right)}^{l-1}$$

where l represents the order of each term in the polynomial, l=0,1,⋅⋅⋅,L, and $b_{l}$ represents the coefficient of each order. $\Delta t_{m}$ is pulse repetition time (PRT). For the convenience of description, polynomial coefficients can be written as a polynomial coefficient vector $b={\left[b_{0},b_{1},\cdot \cdot \cdot ,b_{L}\right]}_{1\times \left(L+1\right)}$. At the same time, the reference distance information used in de-chirp processing can also be obtained from radar measurement information, which is represented as $R_{ref}={\left[R_{ref}\left(1\right),R_{ref}\left(2\right),\cdot \cdot \cdot ,R_{ref}\left(M\right)\right]}_{1\times M}$. It is important to note that, in this instance, a high level of precision for $R_{ref}$ is not necessary. It is only necessary to know exactly what reference distance is used during de-chirp processing; even if there are errors in $R_{ref}$, it will not impact the accuracy of the method proposed. After using $b={\left[b_{0},b_{1},\cdot \cdot \cdot ,b_{L}\right]}_{1\times \left(L+1\right)}$ and $R_{ref}={\left[R_{ref}\left(1\right),R_{ref}\left(2\right),\cdot \cdot \cdot ,R_{ref}\left(M\right)\right]}_{1\times M}$ for joint motion compensation, the ISAR image of the target can be obtained as follows:

(20) $$\begin{array}{l} I\left(k,h\right)=\sum_{m=1}^{M}\exp\left(-j2\pi \frac{hm}{M}\right)\sum_{n=1}^{N}\exp\left(-j2\pi \frac{kn}{N}\right)\\ \;\cdot s\left(n,m\right)\cdot \exp\left[j\frac{4\pi }{c}f_{c}\left(\sum_{l=0}^{L}b_{l}{\left(m\Delta t_{m}\right)}^{l}-R_{ref}\left(m\right)\right)\right]\\ \;\cdot \exp\left[j\frac{4\pi }{c}\gamma \left(\sum_{l=0}^{L}b_{l}{\left(m\Delta t_{m}\right)}^{l}-R_{ref}\left(m\right)\right)n\right]\\ \;\cdot \exp\left[-j\frac{4\pi \gamma }{c}\left(\sum_{l=1}^{L}l\cdot b_{l}{\left(m\Delta t_{m}\right)}^{l-1}\right)\cdot \left(\frac{\sum_{l=1}^{L}l\cdot b_{l}{\left(m\Delta t_{m}\right)}^{l-1}}{c}-1\right)n^{2}\right]\\ \;\cdot \exp\left[j\frac{4\pi }{c}\left(f_{c}\sum_{l=1}^{L}l\cdot b_{l}{\left(m\Delta t_{m}\right)}^{l-1}\right)n\right]+\xi \left(k,h\right) \end{array}$$

If the value of $b={\left[b_{0},b_{1},\cdot \cdot \cdot ,b_{L}\right]}_{1\times \left(L+1\right)}$ is accurately obtained, the high-speed motion and translational motion of the target will be compensated for, and a well-focused ISAR image will be obtained. Hence, the problem of joint motion compensation is essentially an optimal parameter estimation problem. Image entropy [46] is a commonly used evaluation metric in the field of ISAR imaging to measure the quality of image focus. The smaller the entropy, the clearer the image, and the better the focusing performance of the image. Therefore, in this paper, image entropy is chosen as the cost function to implement the optimization of the target motion parameter b.

The ISAR image after joint motion compensation by $\tilde{b}={\left[{\tilde{b}}_{0},{\tilde{b}}_{1},\cdot \cdot \cdot ,{\tilde{b}}_{L}\right]}_{1\times \left(L+1\right)}$, the estimated value of b, can be expressed as

(21) $$\begin{array}{l} I\left(k,h;\tilde{b}\right)=\sum_{m=1}^{M}\exp\left(-j2\pi \frac{hm}{M}\right)\sum_{n=1}^{N}\exp\left(-j2\pi \frac{kn}{N}\right)\\ \;\cdot s\left(n,m\right)\cdot \exp\left[j\frac{4\pi }{c}f_{c}\left(\sum_{l=0}^{L}{\tilde{b}}_{l}{\left(m\Delta t_{m}\right)}^{l}-R_{ref}\left(m\right)\right)\right]\\ \;\cdot \exp\left[j\frac{4\pi }{c}\gamma \left(\sum_{l=0}^{L}{\tilde{b}}_{l}{\left(m\Delta t_{m}\right)}^{l}-R_{ref}\left(m\right)\right)n\right]\\ \;\cdot \exp\left[-j\frac{4\pi }{c}\gamma \left(\frac{{\left(\sum_{l=1}^{L}l\cdot {\tilde{b}}_{l}{\left(m\Delta t_{m}\right)}^{l-1}\right)}^{2}}{c}-\sum_{l=1}^{L}l\cdot {\tilde{b}}_{l}{\left(m\Delta t_{m}\right)}^{l-1}\right)n^{2}\right]\\ \;\cdot \exp\left[j\frac{4\pi }{c}\left(f_{c}\sum_{l=1}^{L}l\cdot {\tilde{b}}_{l}{\left(m\Delta t_{m}\right)}^{l-1}\right)n\right]+\xi \left(k,h\right) \end{array}$$

The image entropy of $I\left(k,h;\tilde{b}\right)$ is related to $\tilde{b}={\left[{\tilde{b}}_{0},{\tilde{b}}_{1},\cdot \cdot \cdot ,{\tilde{b}}_{L}\right]}_{1\times \left(L+1\right)}$, and it can be represented as

(22) $$E_{I}\left(\tilde{b}\right)=\ln S_{I}-\frac{1}{S_{I}}\sum_{k=1}^{N}\sum_{h=1}^{M}{\left|I\left(k,h;\tilde{b}\right)\right|}^{2}\ln{\left|I\left(k,h;\tilde{b}\right)\right|}^{2}$$

where $S_{I}$ is the image intensity that can be expressed as

(23) $$S_{I}=\sum_{k=1}^{N}\sum_{h=1}^{M}{\left|I\left(k,h;\tilde{b}\right)\right|}^{2}$$

The target motion parameter $\tilde{b}={\left[{\tilde{b}}_{0},{\tilde{b}}_{1},\cdot \cdot \cdot ,{\tilde{b}}_{L}\right]}_{1\times \left(L+1\right)}$ can be obtained by minimizing the image entropy $E_{I}\left(\tilde{b}\right)$, expressed as

(24) $$\langle {\hat{b}}_{0},{\hat{b}}_{1},\cdot \cdot \cdot ,{\hat{b}}_{L-1}\rangle =\arg\underset{{\tilde{b}}_{0},\cdot \cdot \cdot ,{\tilde{b}}_{L-1}}{\min}E_{I}\left(\tilde{b}\right)$$

Many algorithms can be used to solve the problem in Equation (24), such as gradient-based methods and intelligent optimization algorithms. However, gradient-based algorithms are complex in calculating derivatives and sensitive to the choice of initial points. Given the inability to provide initial values for the target motion parameters with great precision, the use of gradient-based methods is restricted. To achieve the optimization of target motion parameters, this paper adopts intelligent optimization algorithms to solve the above optimization problems, and the specific steps will be introduced in the next section.

### 3.2. Parameters Optimization Based on RTH-NM

Based on the joint motion compensation optimization model established in Section 3.1, the RTH-NM algorithm is used to estimate the target motion parameters, thereby achieving precise joint motion compensation.

The RTH algorithm is a new nature-inspired metaheuristic optimization algorithm inspired by the red-tailed hawk’s hinting behaviors of a predatory bird. The RTH algorithm exhibits strong robustness and a rapid convergence rate, so it is used to optimize the motion parameters of the space target. The utilization of the RTH algorithm can mitigate the risk of target motion parameter estimation becoming trapped in local optima. Nevertheless, the motion parameters derived from the RTH algorithm often lack sufficient precision, and conducting a highly accurate search for these parameters is time-consuming. To address this, the NM algorithm is applied to enhance the precision of the motion parameters. The specific steps for the RTH-NM algorithm will be described in detail below.

Due to the inability to accurately obtain the target motion parameters, the RTH algorithm needs to be used for a coarse search. The specific steps of an RTH coarse search are as follows:

Step 1 (initialization): The following parameters of the RTH algorithm need to be initialized: the number of red-tailed hawks Q, the maximum number of iterations T, the initial iteration number t, the echo to be compensated $s\left(t_{r},t_{m}\right)$, the radar de-chirp reference distance information $R_{ref}$, the target motion polynomial order L, and the search space for target motion parameters P. In this paper, Q, T, and t are set to 120, 250, and 1, respectively. $s\left(t_{r},t_{m}\right)$ and $R_{ref}$ can be obtained from the radar system. L and P can be obtained from the Two-Line Element Set (TLE) information and $R_{ref}$.

Step 2 (generating the initial position): Based on P, the initial position B of a red-tailed hawk can be obtained, where B is a K-row L+1-column matrix. Calculate the image entropy according to Equation (22), and the optimal position of the red-tailed hawk is $b_{\mathrm{best}}$.

Step 3 (high soaring): The position of the red-tailed hawk is continuously updated, and joint motion compensation is performed using the motion parameters corresponding to each position to obtain the target ISAR image. The image entropy is calculated according to Equation (22), and the position with the minimum entropy is obtained to update the optimal position $b_{\mathrm{best}}$. The position update formula of the red-tailed hawk q is shown in Equation (25):

(25) $$b_{q}^{t}=b_{\mathrm{best}}+\left(b_{\mathrm{mean}}-b_{q}^{t-1}\right)\cdot Levy\cdot \alpha^{t}$$

where $b_{q}^{t}$ represents the position of the red-tailed hawk q at the iteration t, $b_{\mathrm{mean}}$ is the mean position, Levy represents the levy flight distribution function that can be calculated according to Equation (26), and $\alpha^{t}$ denotes the transition factor function that can be calculated according to Equation (28).

(26) $$Levy=s\frac{\mu \cdot \sigma }{{\left|\upsilon \right|}^{\beta -1}}$$

(27) $$\sigma =\left(\frac{\Gamma \left(1+\beta \right)\cdot \sin\left(\pi \beta /2\right)}{\Gamma \left(1+\beta /2\right)\cdot \beta \cdot 2\left(1-\beta /2\right)}\right)$$

where s is a constant (0.01), β is a constant (1.5), and μ and υ are random numbers between 0 and 1.

(28) $$\alpha^{t}=1+\sin\left(2.5+\left(t/T\right)\right)$$

Step 4 (Low soaring): The hawk surrounds the prey by flying much lower to the ground in a spiral line. The position update formula of red-tailed hawk q is shown in Equation (29):

(29) $$b_{q}^{t}=b_{\mathrm{best}}+\left(x_{t}+y_{t}\right)\cdot \left(b_{q}^{t}-b_{\mathrm{mean}}\right)$$

where $x_{t}$ and $y_{t}$ denote direction coordinates, which can be calculated as follows:

(30) $$\left\{\begin{array}{c} x_{t}=R\left(t\right)\cdot \sin\left(\theta \left(t\right)\right)\\ y_{t}=R\left(t\right)\cdot \cos\left(\theta \left(t\right)\right) \end{array}\right.$$

(31) $$\left\{\begin{array}{c} R\left(t\right)=R_{0}\cdot \left(r-t/T\right)\cdot rand\\ \theta \left(t\right)=A\cdot \left(1-t/T\right)\cdot rand \end{array}\right.$$

(32) $$\left\{\begin{array}{c} x_{t}=x_{t}/\max\left|x_{t}\right|\\ y_{t}=y_{t}/\max\left|y_{t}\right| \end{array}\right.$$

where $R_{0}$ denotes the initial value of the radius, which varies from 0 to 1. A is the angle gain, which varies from 5 to 15. rand is a random number between 0 and 1. r is a control gain that varies from 1 to 2.

Step 5 (stooping and swooping): The hawk suddenly stoops and attacks the prey from the best-obtained position in the low soaring stage. The position update formula of red-tailed hawk q is shown in Equation (29):

(33) $$b_{q}^{t}=\psi^{t}\cdot b_{\mathrm{best}}+x_{t}\cdot S1\left(t\right)+y_{t}\cdot S2\left(t\right)$$

where S1 and S2 are step sizes and can be calculated as follows:

(34) $$S1\left(t\right)=b_{k}^{t}-\alpha^{t}\cdot b_{\mathrm{mean}}$$

(35) $$S2\left(t\right)=G^{t}\cdot b_{k}^{t}-\alpha^{t}\cdot b_{\mathrm{best}}$$

where ψ and G are the acceleration and the gravity factors, which can be calculated as follows:

(36) $$\psi^{t}=\sin^{2}\left(2.5-t/T\right)$$

(37) $$G^{t}=2\cdot \left(1-t/T\right)$$

Step 6 (termination condition judgment): If the number of iterations reaches the maximum value T, terminate the search process; otherwise, return to step 3 and continue the search. Finally, the global optimal position $b_{\mathrm{best}}$ is output as the optimal motion parameter, that is, $b_{\mathrm{coarse}}=b_{\mathrm{best}}$.

After the search using the RTH algorithm, the coarse target motion parameters are obtained, but they are not precise enough. Consequently, a refined search is required to obtain the fine target motion parameters. The NM algorithm is used for the precise search of motion parameters, and the specific steps are as follows.

Step 1 (initialization): Use the result $b_{\mathrm{coarse}}$ obtained from the RTH algorithm as the initial input, and initialize *L* + 2 points ${\tilde{b}}_{0},\cdots ,{\tilde{b}}_{l},\cdots ,{\tilde{b}}_{L+1}$, serving as the vertices of the *L* + 1 simplex.

Step 2 (order): Based on the motion parameters corresponding to each vertex ${\tilde{b}}_{l}$, perform joint motion compensation to obtain ISAR images, and calculate the entropy $E_{I}\left({\tilde{b}}_{l}\right)$ of the ISAR images. Then, reorder the vertices according to $E_{I}\left({\tilde{b}}_{l}\right)$ to satisfy $E_{I}\left({\tilde{b}}_{0}\right)\leq E_{I}\left({\tilde{b}}_{2}\right)\leq \cdot \cdot \cdot \leq E_{I}\left({\tilde{b}}_{L+1}\right)$. Check whether the stopping conditions are met.

Step 3 (centroid): Discard the worst point ${\tilde{b}}_{L+1}$, and calculate the centroid of the first L+1 vertices, ${\tilde{b}}_{0}=\frac{1}{L+1}\sum_{l=0}^{L}{\tilde{b}}_{l}$.

Step 4 (reflection): Calculate the reflection point ${\tilde{b}}_{r}={\tilde{b}}_{o}+\rho^{\prime }\left({\tilde{b}}_{o}-{\tilde{b}}_{L+1}\right)$. If $E_{I}\left({\tilde{b}}_{r}\right)$ is better than $E_{I}\left({\tilde{b}}_{L}\right)$ but worse than $E_{I}\left({\tilde{b}}_{0}\right)$, that is, $E_{I}\left({\tilde{b}}_{0}\right)\leq E_{I}\left({\tilde{b}}_{r}\right)\leq E_{I}\left({\tilde{b}}_{L}\right)$, then replace ${\tilde{b}}_{L+1}$ with ${\tilde{b}}_{r}$ to construct a new *L* + 1-simplex and continue with step 2.

Step 5 (expansion): If the reflection point is the optimum, that is, $E_{I}\left({\tilde{b}}_{r}\right)<E_{I}\left({\tilde{b}}_{0}\right)$, then calculate the expansion point ${\tilde{b}}_{e}={\tilde{b}}_{o}+\gamma^{\prime }\left({\tilde{b}}_{r}-{\tilde{b}}_{o}\right)$. If the expansion point is better than the reflection point, that is, $E_{I}\left({\tilde{b}}_{e}\right)<E_{I}\left({\tilde{b}}_{r}\right)$, then replace ${\tilde{b}}_{L+1}$ with ${\tilde{b}}_{e}$ and continue with step 2; otherwise, replace ${\tilde{b}}_{L+1}$ with ${\tilde{b}}_{r}$ and then continue with step 2.

Step 6 (contraction): If $E_{I}\left({\tilde{b}}_{L}\right)\leq E_{I}\left({\tilde{b}}_{r}\right)\leq E_{I}\left({\tilde{b}}_{L+1}\right)$, calculate the contraction point ${\tilde{b}}_{c}={\tilde{b}}_{o}+\alpha^{\prime }\left({\tilde{b}}_{r}-{\tilde{b}}_{o}\right)$. If $E_{I}\left({\tilde{b}}_{c}\right)\leq E_{I}\left({\tilde{b}}_{L+1}\right)$, then replace ${\tilde{b}}_{L+1}$ with ${\tilde{b}}_{c}$ and continue with step 3; otherwise, proceed to step 7. If $E_{I}\left({\tilde{b}}_{r}\right)\geq E_{I}\left({\tilde{b}}_{L+1}\right)$, calculate the inner contraction point ${\tilde{b}}_{cc}={\tilde{b}}_{o}+\alpha^{\prime }\left({\tilde{b}}_{L+1}-{\tilde{b}}_{o}\right)$. If the inner contraction point is better than the worst point, then replace the worst point with ${\tilde{b}}_{cc}$; otherwise, proceed to step 7.

Step 7 (shrink): Use ${\tilde{b}}_{l}={\tilde{b}}_{0}+\sigma^{\prime }\left({\tilde{b}}_{l}-{\tilde{b}}_{0}\right)$ to replace all points except the current optimum point, and then continue with step 2.

In the aforementioned steps, $\rho^{\prime }$, $\gamma^{\prime }$, $\alpha^{\prime }$, and $\sigma^{\prime }$ represent the reflection, expansion, contraction, and reduction coefficients, respectively, with values typically being $\rho^{\prime }=1$, $\gamma^{\prime }=2$, $\alpha^{\prime }=1/2$, and $\sigma^{\prime }=1/2$. After further optimization using the NM algorithm, the precise target motion parameters $b_{\mathrm{fine}}$ can be obtained. By utilizing $b_{\mathrm{fine}}$ for joint motion compensation, a high-quality ISAR image of the target can be achieved.

**Figure 3:**
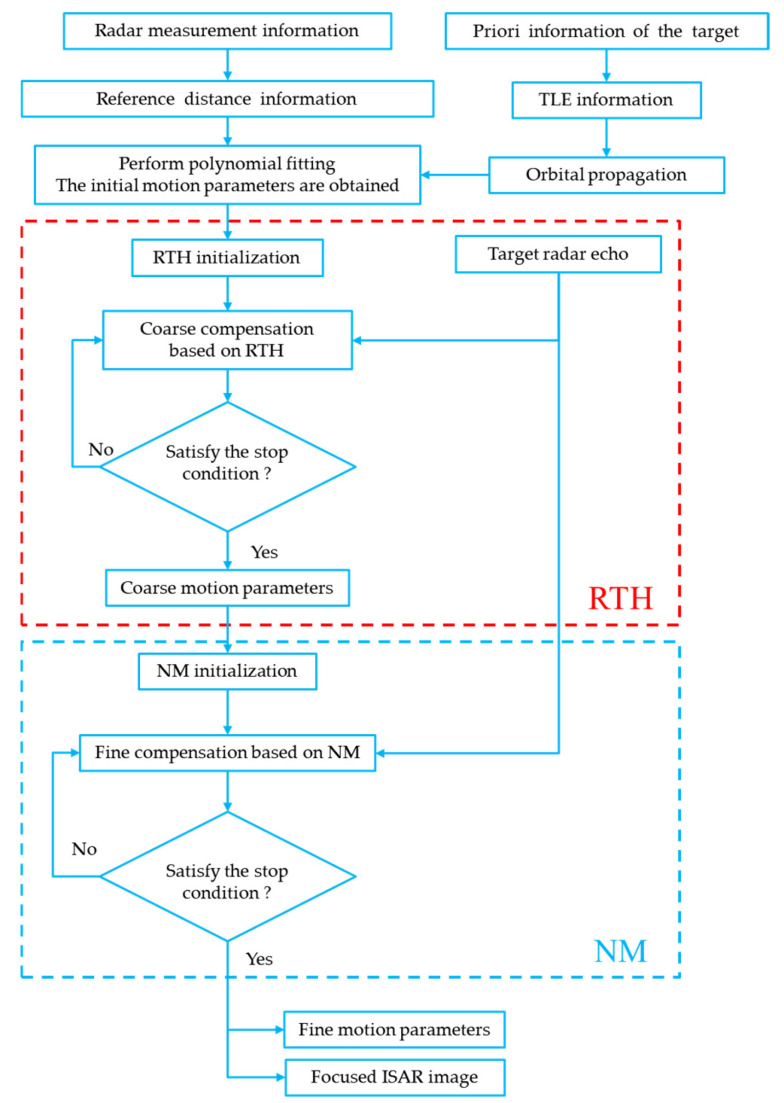
Flowchart of joint motion compensation algorithm based on the RTH-NM algorithm.

In summary, the flowchart of the joint motion compensation algorithm based on the RTH-NM algorithm proposed in this paper is shown in Figure 3.

## 4. Experiment and Discussion

In this subsection, different experiments were designed to demonstrate the performance of the proposed algorithm. The experiments are divided into two types. The first type was conducted using electromagnetic simulation data, and echo simulations were performed using the physical optics (PO) method [47]. The second type was conducted using Yak-42 real measurement data, and the effectiveness and robustness of the proposed algorithm were further verified. The orbital motion of the target in the experiments was calculated from the TLE [48] information, and four different imaging apertures were selected for motion compensation experiments. All the images are generated by the range-Doppler algorithm (RDA); the difference lies in the use of different HSMC algorithms and TMC algorithms. In all experiments, the proposed algorithm is compared with three other algorithms. The first method is the minimum-entropy high-speed motion compensation, minimum-entropy range alignment, and minimum-entropy phase compensation algorithm, referred to as the ME+MERA+MEPA algorithm. The second involves using ICPF for high-speed motion compensation, maximum-contrast range alignment, and maximum-contrast autofocus, referred to as the ICPF+MCRA+MCPA algorithm. The other is the minimum-entropy high-speed motion compensation, minimum-entropy range alignment and sparse Bayesian learning (SBL) minimum-entropy phase compensation algorithm in [18], referred to as the ME+MERA+SBLMEPA algorithm.

### 4.1. Experiments Based on Electromagnetic Simulation

**Figure 4:**
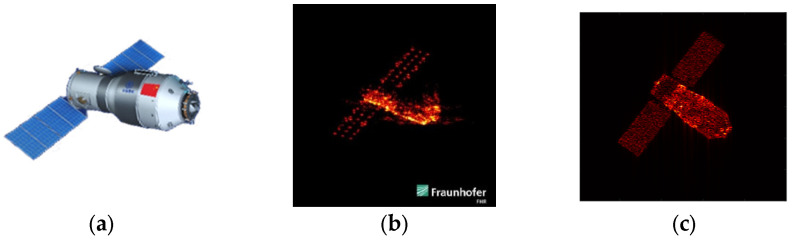
TG-1 Model and its ISAR imaging results. (**a**) The CAD model of TG-1 satellite. (**b**) Real ISAR image. (**c**) EM simulation ISAR image.

Since satellite data are rarely publicly available, the experimental data in this subsection were acquired through electromagnetic simulations based on the Tiangong-1 (TG-1) satellite model. Its three-dimensional model is depicted in Figure 4a. All simulations utilized triangular facet models, segmenting the target surface into tens of thousands of equivalent scatterings. To illustrate the effectiveness of the EM simulation, Figure 4 presents a comparison between the actual ISAR image of TG-1 (Figure 4b) and the EM-simulated ISAR image (Figure 4c). The comparison results indicate that the quality of the generated imagery is comparable to that of the measured ISAR image, thereby supporting the research presented in this paper.

In order to demonstrate the performance of the proposed joint motion compensation algorithm under different motion conditions, different orbit motions are added to the echo. The orbit of the TG-1 satellite is chosen as the simulation orbit, and its TLE [49] is shown in Table 1. The orbit motion of the TG-1 satellite can be calculated by the Simplified General Perturbations 4 (SGP4) model according to the TLE information. The TLE of the TG-1 satellite [49] is listed below.

- 37820U 11053A   16266.35688463   .00025497   00000-0   24137-3 0   9991,
- 37820 042.7662   24.7762 0015742  351.0529  104.2087   15.66280400 28580 8.

**Figure 5:**
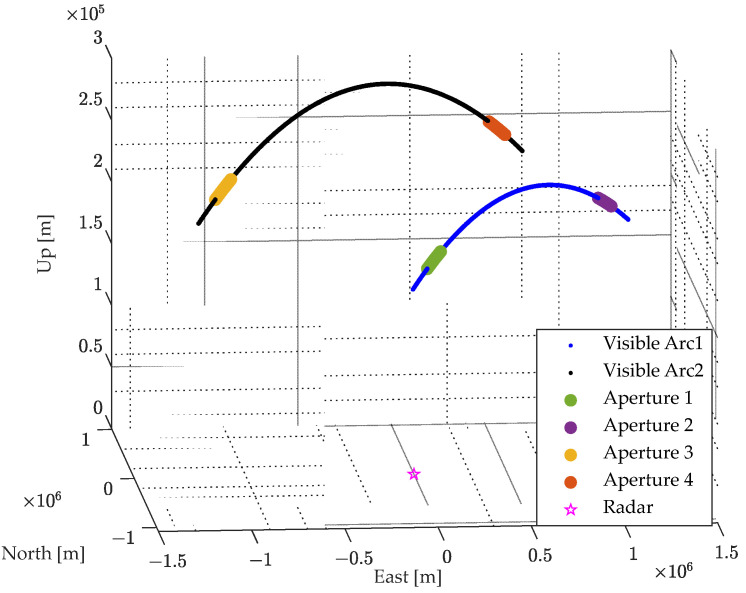
ISAR imaging scene configuration.

The imaging scene configuration is depicted in Figure 5, where the ground-based radar is situated at (29.7° N, 119.8° E), transmitting an LFM signal. The imaging parameters are detailed in Table 1. For echo simulation, four distinct imaging apertures from two visibility arcs is selected, with the respective imaging periods as follows:

- Aperture 1: 22 September 2016 19:0:11~22 September 2016 19:0:25.
- Aperture 2: 22 September 2016 19:3:11~22 September 2016 19:3:25.
- Aperture 3: 22 September 2016 20:35:58~22 September 2016 20:36:12.
- Aperture 4: 22 September 2016 20:39:58~22 September 2016 20:40:12.

Generally, due to the relatively short duration of the imaging CPI, a fourth-order polynomial is sufficient to describe the motion of the target. Therefore, the motion of the target is modeled as a fourth-order polynomial. The coefficients for the polynomials of various orders for imaging apertures 1–4 are shown in Table 2.

**Figure 6:**
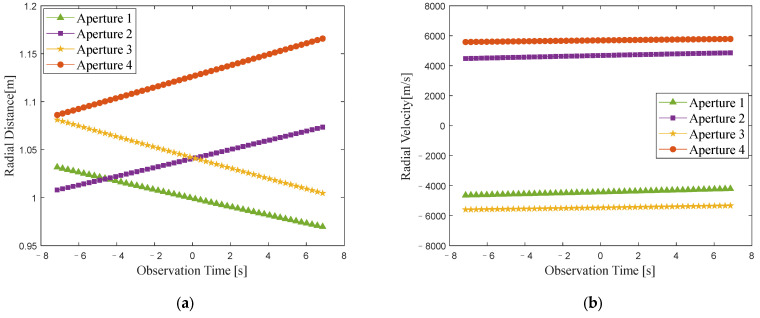
Variations in radial distance and radial velocity within each imaging aperture. (**a**) Variation in the radial distance. (**b**) Variation in the radial velocity.

The variations in the radial distance and radial velocity of the target under different imaging apertures are illustrated in Figure 6. The magnitude of radial velocity varies between 4000 and 6000 m/s, with the maximum change in radial velocity reaching approximately 380 m/s within the CPI. According to the analysis in Section 2, there are high-speed motion errors in the echo, which can cause range profile stretching and range cell shifts. Traditional methods use a single echo to estimate the velocity to operate HSMC, leading to residual errors in high-speed motion that affect the precision of subsequent TMC. This impact is more severe in the case of low SNR. After employing appropriate HSMC methods and TMC methods, the ISAR image of the target can be obtained. It is worth noting that since the raw echo without any compensation cannot be focused for imaging and lacks reference value, we directly present the ISAR imaging results after HSMC and TMC under different imaging apertures.

**Figure 7:**
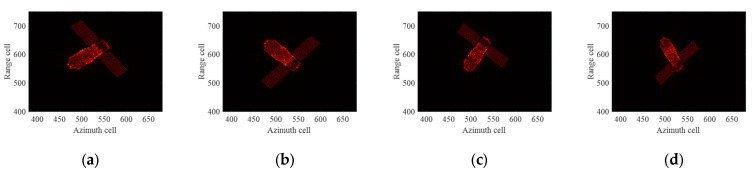
Ideal ISAR images for different imaging apertures. (**a**) Aperture 1; (**b**) Aperture 2; (**c**) Aperture 3; (**d**) Aperture 4.

**Figure 8:**
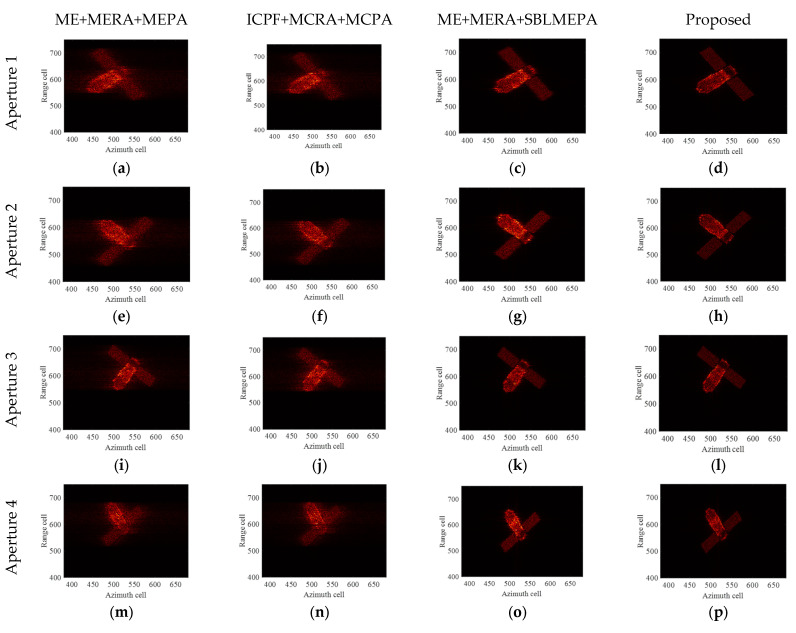
Imaging results of TG-I electromagnetic simulation data under different motion conditions. (**a**–**d**) Imaging results by different algorithms of aperture 1; (**e**–**h**) imaging results by different algorithms of aperture 2; (**i**–**l**) imaging results by different algorithms of aperture 3; and (**m**–**p**) imaging results by different algorithms of aperture 4.

The ideal ISAR images under four different imaging apertures are given as shown in Figure 7. It can be seen that the imaging results under different imaging apertures are significantly different due to the different imaging perspectives and the motion states of the target. The first column of Figure 8 displays the ISAR images obtained after compensation using the ME+MERA+MEPA method, and the second column of Figure 8 shows the ISAR images obtained after compensation using the ICPF+MCRA+MCPA algorithm. It can be observed that the image focusing quality achieved by the ME+MERA+MEPA algorithm is essentially the same as that of the ICPF+MCRA+MCPA algorithm. Due to residual errors in HSMC, both images are slightly defocused and blurred. The third column of Figure 7 displays the focused ISAR images obtained by the ME+MERA+SBLMEPA algorithm. Due to the phase compensation based on sparse Bayesian entropy minimization adopted in the ME+MERA+SBLMEPA algorithm, the defocusing and blurring of the images are eliminated to a certain extent, and the focusing quality of the images is higher than that of the images obtained by the ME+MERA+MEPA algorithm and the ICPF+MCRA+MCPA algorithm. The fourth column of Figure 8 displays the focused ISAR images obtained by the proposed algorithm. It can be seen that the proposed algorithm obtains a better focused image due to joint compensation for high-speed motion and translational motion, which eliminates the residual error. In order to quantify the imaging results, the entropy of the images after motion compensation by different algorithms is given, as shown in Table 3. It can be seen from Table 3 that compared with the ME+MERA+MEPA algorithm, the ICPF+MCRA+MCPA algorithm, and the ME+MERA+SBLMEPA algorithm, the image entropy obtained by the proposed algorithm is smaller and closer to the ideal image. Therefore, the proposed method has better motion compensation performance.

**Figure 9:**
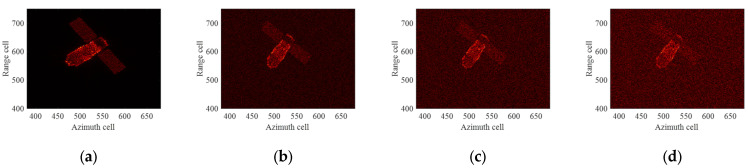
Ideal ISAR images under different SNRs. (**a**) SNR = 0 dB; (**b**) SNR = −5 dB; (**c**) SNR = −9 dB; (**d**) SNR = −13 dB.

**Figure 10:**
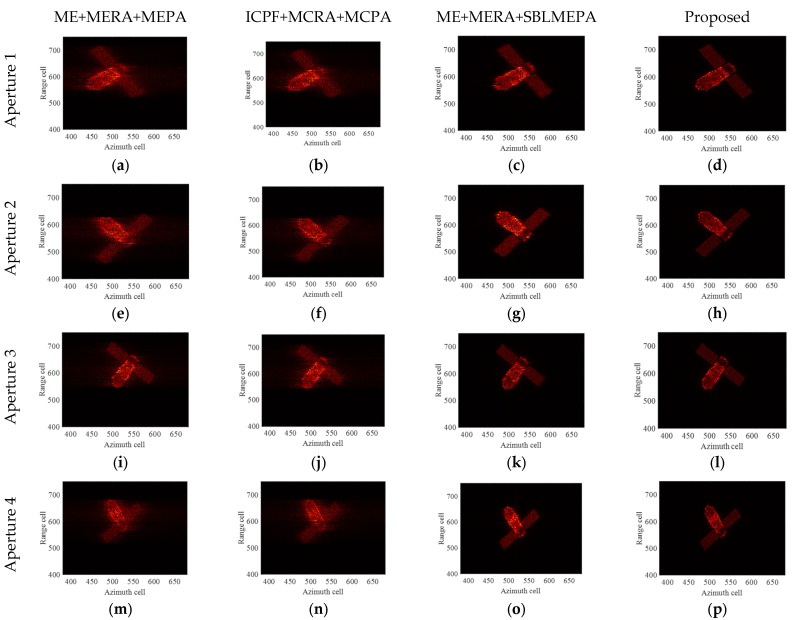
Imaging results of TG-I electromagnetic simulation data under different SNRs. (**a**–**d**) ISAR imaging results obtained by different algorithms, SNR = 0 dB; (**e**–**h**) ISAR imaging results obtained by different algorithms, SNR = −5 dB; (**i**–**l**) ISAR imaging results obtained by different algorithms, SNR = −9 dB; and (**m**–**p**) ISAR imaging results obtained by different algorithms, SNR = −13 dB.

To verify the performance of the proposed algorithm under low-SNR conditions, complex Gaussian white noise is added to the electromagnetic simulation data to produce different SNRs (from 0 dB to −13 dB). The electromagnetic simulation data and orbital data of aperture 3 are chosen for the experiment. Figure 9 presents the ideal images under different SNRs. The ISAR images after compensation by different motion compensation algorithms are shown in Figure 10. The first and second columns show the images obtained after motion compensation using the ME+MERA+MEPA algorithm and the ICPF+MCRA+MCPA algorithm. The third and fourth columns present the images obtained using the ME+MERA+SBLMEPA algorithm and the proposed algorithm. In Figure 10, it is noteworthy that due to intense noise, there are residual errors in HSMC, and the correlation of the HRRP is reduced, leading to a decrease in the precision of TMC. As a result, the images obtained by the traditional motion compensation algorithms are of poor quality with artifacts in the images. When the SNR is less than −5 dB, the images begin to defocus, and when the SNR is less than −9 dB, all traditional motion compensation algorithms fail, making focused imaging virtually impossible. Compared to the other two algorithms, the ME+MERA+SBLMEPA algorithm performs better, but it also has limited performance when the SNR is lower than −9 dB. This is due to the fact that at this time, the intense noise will result in residual errors in motion compensation. Consequently, it is not possible to enhance the image focusing effect by improving the accuracy of PA. In contrast, the proposed algorithm can achieve precise motion compensation and achieve better focusing results when the SNR is not lower than −13 dB. Table 4 provides the entropy of motion-compensated images after applying different algorithms at various SNRs. Table 4 indicates that the proposed algorithm demonstrates the best performance, achieving the lowest image entropy compared to other algorithms and coming closer to the ideal image entropy. Therefore, the motion compensation performance and robustness of the proposed algorithm are further demonstrated.

Experiments show that the proposed algorithm is unable to compensate for target motion when the SNR is below −13 dB and decreases further, resulting in severe blurring of the compensated images. This is because the relationship between focus quality and image entropy is not consistent when the data contain very strong noise, and the image entropy depends almost entirely on the strong noise, independent of the joint motion compensation. In this case, a higher SNR gain can be obtained using more pulses for the imaging process, and then well-focused images can be generated by the proposed algorithm. Overall, the algorithm shows good robustness when dealing with noise.

### 4.2. Experiments Based on Measured Yak-42 Data

In order to verify the performance of the proposed algorithm on the measured data, this section conducts a performance analysis of the proposed algorithm using the Yak-42 measured data. The orbital motion with different apertures and different noises are added to the data, and different TSMC and TMC algorithms are executed. The YAK-42 aircraft dataset was recorded by a C-band ISAR experimental system with a center frequency of 5.52 GHz and a bandwidth 400 MHz. The de-chirp sampling rate is 10 MHz.

**Figure 11:**
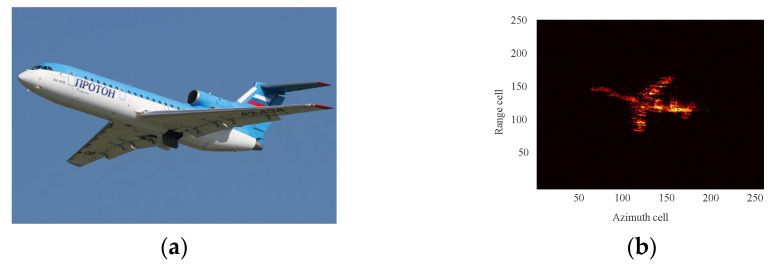
Optical image and ideal ISAR imaging of Yak-42 airplane. (**a**) Optical image; (**b**) ISAR image.

**Figure 12:**
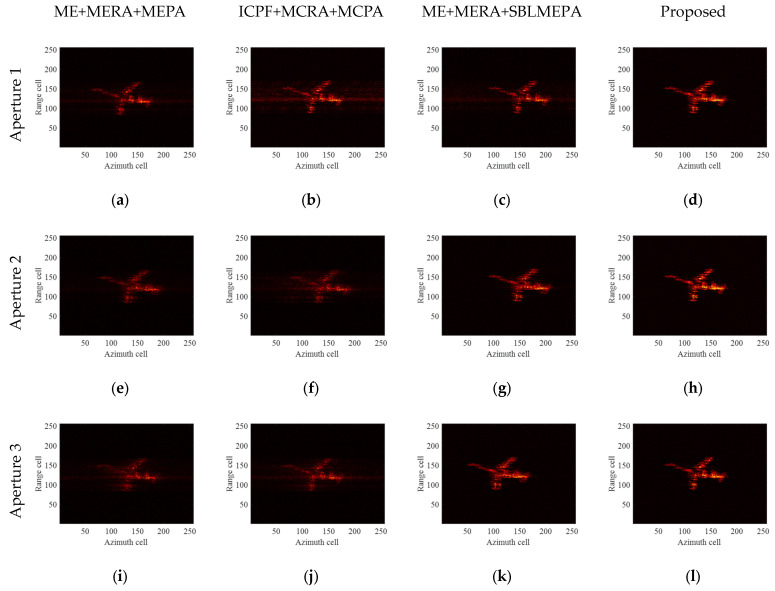

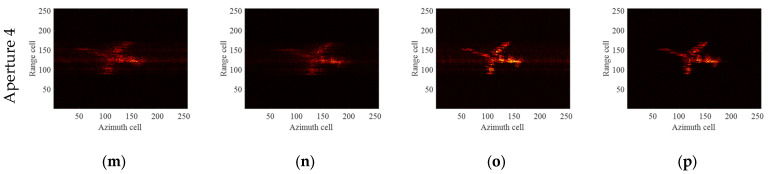
Imaging results of Yak-42 measured data under different motion conditions. (**a**–**d**) Imaging results by different algorithms of aperture 1; (**e**–**h**) imaging results by different algorithms of aperture 2; (**i**–**l**) imaging results by different algorithms of aperture 3; and (**m**–**p**) imaging results by different algorithms of aperture 4.

The image of the Yak-42 aircraft is shown in Figure 11a, and its ideal ISAR image is shown in Figure 11b. The velocity of the aircraft is relatively small compared to that of space targets, so the effect of speed on the echo can be essentially ignored. Therefore, the motions of different imaging apertures from Table 2 were incorporated into the raw radar echo of YAK-42. As with the previous experiment, different algorithms are used for HSMC and TMC, and the corresponding imaging results are shown in Figure 12.

It can be clearly seen from Figure 12 that compared with the ME+MERA+MEPA algorithm, the ICPF+MCRA+MCPA algorithm, and the ME+MERA+SBLMEPA algorithm, no matter which aperture movement is added to the YAK-42 measured data, the proposed algorithm can obtain significantly clearer images. On the contrary, the images obtained by the ME+MERA+MEPA algorithm and the ICPF+MCRA+MCPA algorithm have poor focusing quality, the images are defocused, and there are many artifacts. Compared with the ME+MERA+MEPA algorithm and the ICPF+MCRA+MCPA algorithm, the focusing quality of the images obtained by utilizing the ME+MERA+SBLMEPA algorithm has been improved, but there is still a small amount of artifacts and localized defocusing. In order to better show the advantages of the proposed algorithm, Table 5 gives the image entropy after motion compensation using different HSMC and TMC algorithms. It can be seen that the image entropy compensated for by the proposed algorithm is the smallest, and it still has the best performance on the measured data.

**Figure 13:**
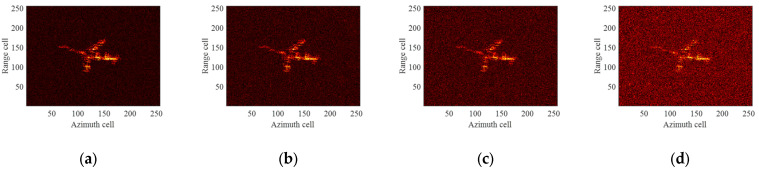
Ideal ISAR images under different SNRs. (**a**) SNR = 0 dB; (**b**) SNR = −3 dB; (**c**) SNR = −6 dB; (**d**) SNR = −10 dB.

**Figure 14:**
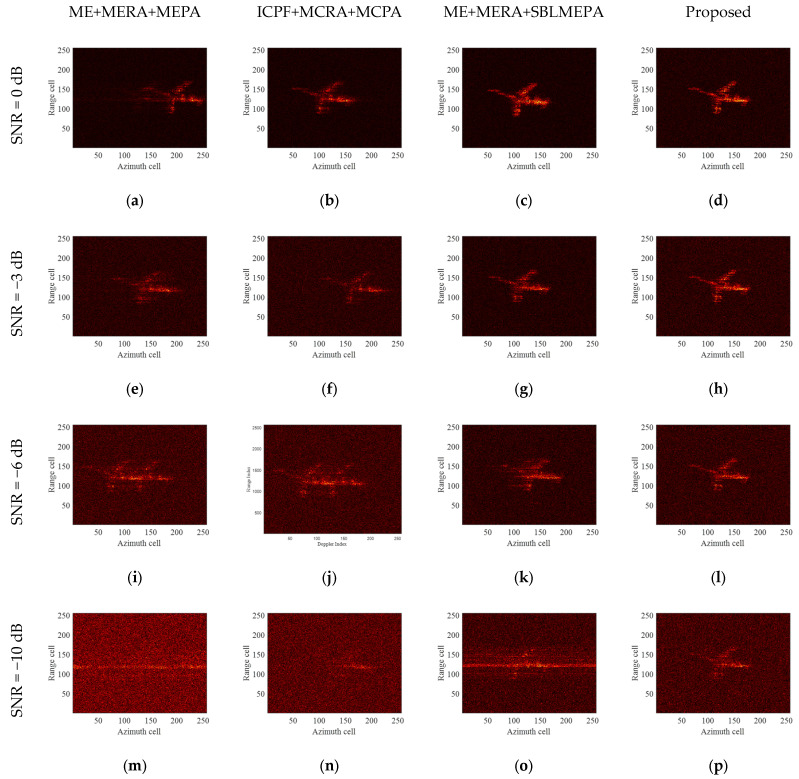
Imaging results of Yak-42 measured data under different SNRs. (**a**–**d**) ISAR imaging results obtained by different algorithms, SNR = 0 dB; (**e**–**h**) ISAR imaging results obtained by different algorithms, SNR = −3 dB; (**i**–**l**) ISAR imaging results obtained by different algorithms, SNR = −6 dB; and (**m**–**p**) ISAR imaging results obtained by different algorithms, SNR = −10 dB.

In order to verify the performance of the proposed algorithm under different noises, noises with different SNR are added to the YAK-42 data, and the motion parameters of the target are consistent with those of aperture 3. The ideal ISAR images under different SNRs are shown in Figure 13. The imaging results after using different HSMC and TMC algorithms are shown in Figure 14. Different columns are the imaging results obtained using different motion compensation algorithms. By comparison, it can be found that the ME+MERA+MEPA algorithm and the ICPF+MCRA+MCPA algorithm are basically unable to image when the SNR is lower than −3 dB because the Yak-42 measured data are more complex than the electromagnetic simulation data. The motion compensation performance of the ME+MERA+SBLMEPA algorithm on Yak-42 measured data is somewhat better than the other two conventional algorithms, but it also fails to focus the image when the SNR is below −5 dB. On the contrary, the proposed algorithm obtains a well-focused image at a low SNR (no less than −10 dB). Similarly, the entropy of the compensated images under different SNRs is shown in Table 6. It can be seen that the performance of the proposed method is the best.

The complexity of the algorithm is also one of the factors that need to be considered in the actual processing. Table 7 gives a comparison of the operation time of the proposed algorithm with other algorithms. The CPU time is obtained by Matlab 2021a coding using a personal computer equipped with an Intel Core i5-6200 U 2.4 GHz processor and 16 GB of memory. A total of 200 Monte Carlo simulations were performed and averaged. It can be seen from Table 7 that the proposed algorithm takes longer than the other three algorithms. This is because the proposed algorithm accurately estimates the target motion parameters, and the complexity of the RTH algorithm itself is high, resulting in a longer operation time. However, compared with a shorter time to obtain defocused images, if high-quality ISAR images can be obtained, it is acceptable to use a relatively long time. At the same time, if the computing performance of the computer can be enhanced, the time consumption of this algorithm will be reduced further.

## 5. Conclusions

The high-speed motion of a space target will lead to a stretch in HRRP and affect the accuracy of the subsequent TMC. Under low-SNR conditions, the residual error of high-speed motion may even lead to the failure of traditional TMC algorithms. A new parametric joint motion compensation algorithm is proposed for the ISAR imaging of space targets under low-SNR conditions. In this paper, a joint compensation algorithm for high-speed motion and translational motion is innovatively carried out, which reduces the influence of residual error of high-speed motion on TMC. In this algorithm, the target motion in a CPI is modeled as a high-order polynomial, and a parameterized minimum entropy optimization model is established. By making full use of the TLE information and radar measurement information of the target, the RTH-NM algorithm is used to quickly and accurately search the motion polynomial coefficients of the target, so as to realize joint compensation for the high-speed motion and translation motion of the target and to obtain high-quality ISAR imaging. The algorithm has good noise robustness and can accurately compensate for the high-speed motion and translation motion of the target under low-SNR conditions. Electromagnetic simulation data and measured data experiments verify the effectiveness of the proposed algorithm. However, our algorithm is more complex than the traditional algorithm, which is our next improvement. Meanwhile, the performance of the proposed algorithm is limited under the conditions of target acceleration maneuvering and sparse apertures. Our future work will focus on these issues.

## Figures and Tables

**Table 1:** Radar parameters of the simulation.

Center Frequency	Pulse Repetition Frequency	Pulse Width	Band Width	Sample Frequency
12 GHz	80 Hz	200 us	2 GHZ	10 MHz

**Table 2:** Motion parameters of the target in various apertures.

	$b_{0}$	$b_{1}$	$b_{2}$	$b_{3}$	$b_{4}$
Aperture 1	9.993 × 10^5^	−4432	15.570	0.0696	6.144 × 10^−6^
Aperture 2	1.041 × 10^6^	4688	13.660	−0.0627	1.809 × 10^−6^
Aperture 3	1.042 × 10^6^	−5469	9.477	0.0504	9.588 × 10^−5^
Aperture 4	1.126 × 10^6^	5694	7.279	0.0383	7.763 × 10^−5^

**Table 3:** Entropy of images acquired by different algorithms.

	Aperture 1	Aperture 2	Aperture 3	Aperture 4
Ideal Image	11.9732	11.9087	11.8467	11.9437
ME+MERA+MEPA	11.9983	12.1623	12.0865	12.0051
ICPF+MCRA+MCPA	11.9918	11.9217	11.8743	11.9661
ME+MERA+SBLMEPA	11.9897	11.9203	11.8649	11.9607
Proposed	11.9819	11.9102	11.8572	11.9543

**Table 4:** Entropy of images under different SNRs.

	0 dB	−5 dB	–9 dB	–13 dB
Ideal Image	13.8573	14.0153	14.2977	14.7418
ME+MERA+MEPA	13.8675	14.1963	14.8536	15.9636
ICPF+MCRA+MCPA	13.9324	14.1879	14.8411	15.9358
ME+MERA+SBLMEPA	13.8653	14.1527	14.7369	15.9175
Proposed	13.8627	14.0738	14.3683	15.0156

**Table 5:** Entropy of images acquired by different algorithms using Yak-42 measured data.

Image Entropy				
Ideal Image	7.4752			
	Aperture 1	Aperture 2	Aperture 3	Aperture 4
ME+MERA+MEPA	7.4868	7.4937	7.4916	7.5125
ICPF+MCRA+MCPA	7.4965	7.4887	7.4865	7.4879
ME+MERA+SBLMEPA	7.4835	7.4856	7.4827	7.5025
Proposed	7.4775	7.4802	7.4794	7.4785

**Table 6:** Entropy of images with different SNRs based on Yak-42 measured data.

Image Entropy vs. SNR				
SNR	0 dB	–3 dB	–6 dB	–10 dB
Ideal Images	9.5783	10.3027	10.5897	10.6349
ME+MERA+MEPA	9.6208	10.3853	10.6753	10.7641
ICPF+MCRA+MCPA	9.7379	10.3769	10.6561	10.7538
ME+MERA+SBLMEPA	9.6135	10.3674	10.6429	10.7363
Proposed	9.5825	10.3481	10.6135	10.6995

**Table 7:** Computation time comparison.

Algorithms	Computation Time (s)
ME+MERA+MEPA	210
ICPF+MCRA+MCPA	305
ME+MERA+SBLMEPA	253
Proposed	691

## Data Availability

Data are available on request from the authors.
